# Derivation and validation of a nomogram incorporating modifiable lifestyle factors to predict development of colorectal adenomas after negative index colonoscopy

**DOI:** 10.1038/s41598-024-62348-w

**Published:** 2024-05-21

**Authors:** Mingqian Yu, Yiben Ouyang, Zhen Yuan, Shuyuan Wang, Wenwen Pang, Suying Yan, Xinyu Liu, Wanting Wang, Ben Yi, Qiurong Han, Yao Yao, Yanfei Liu, Jiachun Song, Tianhao Chu, Zhiqiang Feng, Qinghuai Zhang, Xipeng Zhang, Chunze Zhang

**Affiliations:** 1 School of Medicine, Nankai University, Tianjin, 300071 China; 2 Department of Colorectal Surgery, Tianjin Union Medical Center, Tianjin, 300121 China; 3 School of Integrative Medicine, Tianjin University of Traditional Chinese Medicine, Tianjin, 301617 China; 4 Tianjin Medical University, Tianjin, 300041 China; 5 Department of Clinical Laboratory, Tianjin Union Medical Center, Tianjin, 300121 China; 6 The Institute of Translational Medicine, Tianjin Union Medical Center, Tianjin, 300121 China; 7 Tianjin Institute of Coloproctology, Tianjin, China

**Keywords:** Colonoscopy, Gastrointestinal diseases, Colorectal cancer

## Abstract

This retrospective cohort study aimed to identify baseline patient characteristics involving modifiable lifestyle factors that are associated with the development of colorectal adenomas, and establish and validate a nomogram for risk predictions among high-risk populations with negative index colonoscopy. A total of 83,076 participants who underwent an index colonoscopy at the Tianjin Union Medical Center between 2004 and 2019 were collected. According to meticulous inclusion and exclusion criteria, 249 subjects were enrolled and categorized into the primary and validation cohorts. Based on the primary cohort, we utilized the LASSO-Cox regression and the univariate/multivariate Cox proportional hazards (Cox-PH) regression parallelly to select variables, and incorporated selected variables into two nomogram models established using the multivariate Cox-PH regression. Comparison of the Akaike information criterion and the area under the receiver operating characteristic curve of the two models demonstrated that the nomogram model constituted by four covariates retained by the LASSO-Cox regression, including baseline age, body mass index, physical activity and family history of colorectal cancer (CRC) in first-degree relatives, performed better at predicting adenoma-free survival probabilities. Further validation including the concordance index, calibration plots, decision curve analysis and Kaplan–Meier survival curves also revealed good predictive accuracy, discriminating ability, clinical utility and risk stratification capacity of the nomogram model. Our nomogram will assist high-risk individuals with negative index colonoscopy to prevent colorectal adenoma occurrence and CRC morbidity with improved cost-effectiveness.

## Introduction

Colorectal cancer (CRC) ranks third in terms of incidence among cancers and remains the second leading cause of cancer-related mortality globally, with more than 1.06 million new CRC cases and 515,000 deaths estimated to occur in men, and more than 0.86 million new CRC cases and 419,000 deaths estimated to occur in women, based on the GLOBOCAN 2020 estimates^1^. In the past few decades, China has undergone a convergence toward the common cancer profiles in developed countries, characterized by the high incidence and mortality of CRC, in parallel with an escalating adult population size, swift population aging, socioeconomic developments and especially, accumulative exposure to lifestyle risk factors that prevail in western countries^1–3^.

The development of most sporadic CRC follows the adenoma-carcinoma sequence^4^, and colorectal adenomas are neoplastic lesions of the large bowel that are widely recognized as precursors for the vast majority of CRC cases^5^. Earlier detection of CRC can improve the prognosis prominently since the 5-year relative survival rate is up to 90% when the lesions remain localized^6^. Interrupting this adenoma-carcinoma sequence with population-based screening programs is thus warranted to boost timely detection and early prevention of CRC.

A series of screening modalities for CRC are currently available, including fecal immunochemical test (FIT), colonoscopy and flexible sigmoidoscopy, etc. Among them, colonoscopy is endorsed as the gold standard^7,8^. Considering the large population and the overwhelming constraints of healthcare resources, it is impractical for China to provide a “one-size-fit-all” screening service across each province and county. Nowadays, most CRC screening programs in China adopt a two-step screening strategy, which first identifies individuals with a positive result of either high-risk factor questionnaire (HRFQ) or FIT as high-risk individuals, and subsequently recommends them to undergo colonoscopy using repeat screening^9^. However, the effectiveness of current risk stratification systems has been limited by incremental healthcare expenditures^10^, insufficient endoscopic capacity^11^ and relatively low participation rates^7,8^. In the era of precision medicine, the exploitation of state-of-the-art techniques and suitable risk-adapted screening strategies based on sophisticated risk prediction models which incorporate multisectoral risk factors is of paramount significance for implementing individualized intervention^8,12^.

Because of the general absence of pertinent symptoms, the frequency of colorectal adenomas has commonly been calculated as prevalence rates rather than incidence rates in different populations. The prevalence rates are usually computed based on each participant with a positive index colonoscopy. Nevertheless, actual adenoma incidence rates can be described only by follow-up colonoscopies in each individual whose initial colonoscopy was negative^13,14^. Swelling ranks of studies have reiterated that various modifiable lifestyle factors including smoking status stas^15,16^, alcohol consumption^17,18^, and physical activity^19,20^ are independently and significantly correlated with the presence and development of colorectal adenomas. However, current risk scoring models in predicting risks of developing colorectal neoplasms predominantly rely on demographic and clinicopathological features and often ignore some well-documented lifestyle factors. These unmeasured confounders may bias the observed correlation and the weighing of collected risk factors, thus jeopardizing the performance and utility of models^21^.

In the present study, we embarked on a retrospective cohort study based on the first surveillance colonoscopies that high-risk individuals underwent after negative index colonoscopy. We incorporated various modifiable lifestyle patterns with demographic and clinical characteristics of subjects to establish a nomogram, which could reduce statistical predictive models into an intuitive numerical estimate of the probability of certain event^22^, for predicting risks of developing colorectal adenomas within different time periods for each subject. In a gesture to facilitate risk stratification, our model will provide ponderable reference for the primary and secondary prevention of colorectal adenomas and CRC.

## Methods

### Study design and population

#### Screening of high-risk individuals

In conformity with the two-step screening strategy, each participant was required to first complete the HRFQ and then undergo FIT. The HRFQ was mainly comprised of four questions: (1) with or without a personal history of cancer; (2) with or without a personal history of colorectal adenomas; (3) with or without a family history of CRC in first-degree relatives (FDR); (4) with or without a personal history of two or more items of the following conditions: chronic constipation, chronic diarrhea, mucous bloody stools, adverse life events, chronic appendicitis or appendectomy, and chronic cholecystitis or cholecystectomy. An affirmative answer to any of the four questions was defined as a positive result of HRFQ. Participants with a positive result of either HRFQ or FIT were classified into the high-risk group while the remaining were categorized into the average-risk group. Screening physicians would intensively recommend high-risk participants to undergo colonoscopy examination. According to the findings of index colonoscopy, high-risk individuals were subsequently recommended to undergo surveillance colonoscopies in conformity with authoritative guidelines and were followed up by trained investigators.

#### Inclusion of the study population

Based on electronic medical records, the first-recorded colonoscopy that each high-risk individual underwent within our study period was considered as the index colonoscopy. The index colonoscopy was designated as a positive colonoscopy if there existed any colorectal adenoma, whereas a negative index colonoscopy denoted that there existed no abnormalities.

A total of 83,076 participants who underwent an index colonoscopy with available records at the Tianjin Union Medical Center between March 16, 2004, and December 31, 2019, were collected. Individuals aged under 40 years or over 75 years were considered ineligible (n = 2655). Individuals with a personal history of cancer (n = 1506) or colorectal adenomas (n = 3562) before the index colonoscopy were also rejected. Moreover, 2410 subjects whose index colonoscopies were considered unqualified were excluded. Among the remaining 72,943 participants, subjects who were diagnosed with other colorectal diseases including non-adenomatous colorectal polyps, CRC, inflammatory bowel disease (Crohn’s disease or ulcerative colitis), familial adenomatous polyposis and P-J syndrome, etc. at the index colonoscopy were excluded (n = 6916). 33,752 subjects diagnosed with colorectal adenomas at the index colonoscopy were also dismissed. Among the remaining 32,275 subjects who had negative index colonoscopy, those who underwent at least one surveillance colonoscopy within the study period with the first surveillance colonoscopy being conducted later than 6 months after the index colonoscopy were enrolled into this retrospective cohort study (n = 571). As discovered by the first surveillance colonoscopy and confirmed by pathological reports, 55 subjects developed aforementioned other colorectal diseases, and 290 subjects had newly developed colorectal adenomas, while 226 subjects had no abnormalities. Each subject whose first surveillance colonoscopy was considered unqualified was eliminated (n = 7 for the negative group and n = 9 for the positive group). Individuals who provided unreliable or incomplete baseline information related to any of the studied factors were also excluded (n = 65 for the negative group and n = 186 for the positive group).

Ultimately, 249 qualified subjects consisted of 95 persons in the occurrence group and 154 persons in the non-occurrence group were categorized into the primary cohort (n = 174) and the validation cohort (n = 75) according to the chronological order of dates of index colonoscopy at a ratio of 7:3.

### Colonoscopy examination and pathological diagnosis

All colonoscopies were performed by a group of skilled endoscopists who were certified by the medical facility’s Endoscopy Committee and had at least five years of experience. Only complete colonoscopies with successful cecal intubation, photo documentation of caecal landmarks, adequate bowel preparation and a withdrawal time > 6 min were considered as qualified colonoscopies and included for analysis according to up-to-date clinical guidelines^23–26^. The endoscopists removed all polyps detected, and forwarded them to the pathological laboratory, where the histology of polyps was further confirmed by pathologists. Pathological diagnosis was made by two experienced pathologists separately, and only concordant results were adopted.

### Outcomes and definitions

The outcome in this study was adenoma-free survival (AFS). Similar to previous literature, we excluded subjects with their first surveillance colonoscopy being conducted within six months after negative index colonoscopy, because any adenoma which was detected at subsequent colonoscopies performed within six months after the index colonoscopy was perceived as a missing adenoma that had already existed at the index colonoscopy^27,28^.

Colorectal polyps were classified as neoplastic adenomatous polyps (tubular, tubular villous or villous) or non-neoplastic polyps (hyperplastic, hamartomatous or inflammatory) in accordance with standard criteria^29^. A polyp with a mixed histology (e.g., both hyperplastic and adenomatous components) was considered as an adenoma. BMI was calculated as weight in kilograms divided by height in meters squared. Participants were categorized into never-smokers and smokers according to their smoking status, with the former including subjects who never smoke, and the latter comprised of subjects who smoke currently (smoking at least one pack of cigarettes per week over the last year) or in the past. Participants were also classified into never-drinkers and drinkers in terms of alcohol consumption, with the former including subjects who never drank, and the latter comprised of subjects who drank occasionally, frequently or even daily over the last year. Physical activity was sorted into regular activity and physical inactivity estimated by the frequency of exercise. The former denotes at least 30 min of exercise more than once weekly over the last year, and the latter is defined otherwise. Chronic constipation refers to constipation lasting for more than two months per year in the last two years. Chronic diarrhea refers to diarrhea lasting for more than one week each time, with a cumulative course of more than three months per year in the last two years.

### Formulation and validation of the nomogram

Ten variables encompassing demographic and clinical characteristics of subjects as well as various modifiable lifestyle patterns were chosen as candidate predictors for colorectal adenoma occurrence anchored in previous literature, including baseline age^7,16,30,31^, BMI^32^, gender^30,33^, smoking status^15,16^, alcohol consumption^17,18^, physical activity^19,20^, family history of CRC in FDR^7,34^, history of chronic constipation^35^, history of chronic diarrhea^31,36^, and history of chronic appendicitis or appendectomy^36^. Two methods were deployed to screen out putative risk predictors from the ten variables for construction of nomogram models based on the primary cohort. Firstly, using the LASSO-Cox method (Method-1), variables with non-zero coefficients retained by the LASSO regression were entered into the multivariate Cox proportional hazards (Cox-PH) regression*.* Variables with *p* < 0.05 were considered as independent predictors and incorporated into a nomogram model (Nomogram-1) constructed using the multivariate Cox-PH regression. Secondly, using the univariate/multivariate Cox-PH regression (Method-2), variables with *p* < 0.05 as revealed by the univariate Cox-PH regression were entered into the multivariate Cox-PH regression analysis. The independent predictors determined by the multivariate analysis were absorbed into Nomogram-2 formulated using the multivariate Cox-PH regression. Performance of the two nomograms was compared by calculating the Akaike information criterion (AIC) and the area under the receiver operating characteristic (ROC) curve (AUC) values. Anchored in the concept of entropy, AIC is a measure of the goodness of fit of regression models. A lower AIC value denotes a better model fit^37^. Meanwhile, a higher AUC value signifies a better discriminative power of the model^38^. Ultimately, the nomogram with lower AIC and higher AUC values was selected to predict risks of colorectal adenoma occurrence. The proportional hazards (PH) assumption, which is the underlying premise for the Cox-PH model, was tested by Schoenfeld residual plots and deviance residual before performing the univariate or multivariate Cox-PH regression analysis. Additionally, multicollinearity among predictors in the multivariate Cox-PH regression analysis was assessed using variation inflation factor (VIF), and only predictors with VIF values < 5 were entered^39^.

To evaluate the performance of the nomogram, we calculated the risk score for each subject and computed the optimal cut-off value of the nomogram in the primary cohort and the validation cohort respectively. The utility of the model was judged by the sensitivity (SE), specificity (SP), positive predictive value (PPV), negative predictive value (NPV), positive likelihood ratio (PLR) and negative likelihood ratio (NLR) for subject stratification into the occurrence group and the non-occurrence group based on the optimal cut-off values in both cohorts. The accuracy of the selected nomogram was appraised via two approaches. Firstly, the predictive accuracy for individual outcomes (discrimination) was determined using the concordance index (C-index) values^38^. Secondly, the precision of point estimates of the survival function (calibration) was assessed by comparing the predicted and actual probability of outcomes. Bootstraps with 1000 resamples were employed for these activities. Moreover, clinical utility of the nomogram was judged by the decision curve analysis (DCA). To verify the risk stratification ability of the nomogram, subjects were classified into the high-risk group and the low-risk group according to the optimal cut-off value of risk scores in each cohort. The Kaplan–Meier (KM) survival curves with log-rank tests were utilized to assess whether the survival outcomes were significantly different between the two groups.

### Statistical analysis

The Kolmogorov–Smirnov test was employed to examine the distribution of continuous variables. Continuous variables with normal distribution were presented as mean with standard error and compared using the Student’s *t*-test. Continuous variables with non-normal distribution were presented as median with interquartile ranges (IQRs) and compared using the Wilcoxon–Mann–Whitney test. Categorical variables were exhibited as whole numbers with proportions and compared using the Pearson Chi-square test or continuity correction Chi-square test. Statistical analysis was performed with SPSS software (version 19.0, SPSS Inc., Chicago, IL, USA) and R software (version 4.0.2, http://www.r-project.org). *P* < 0.05 was considered as statistically significant.

### Ethics approval and consent to participate

This study was conducted following the Declaration of Helsinki and approved by the Ethics Committee of Tianjin Union Medical Center. All participants provided their written informed consent and their privacy and identities were fully protected in the manuscript.

## Results

### Baseline characteristics of the study population

**Figure 1:**
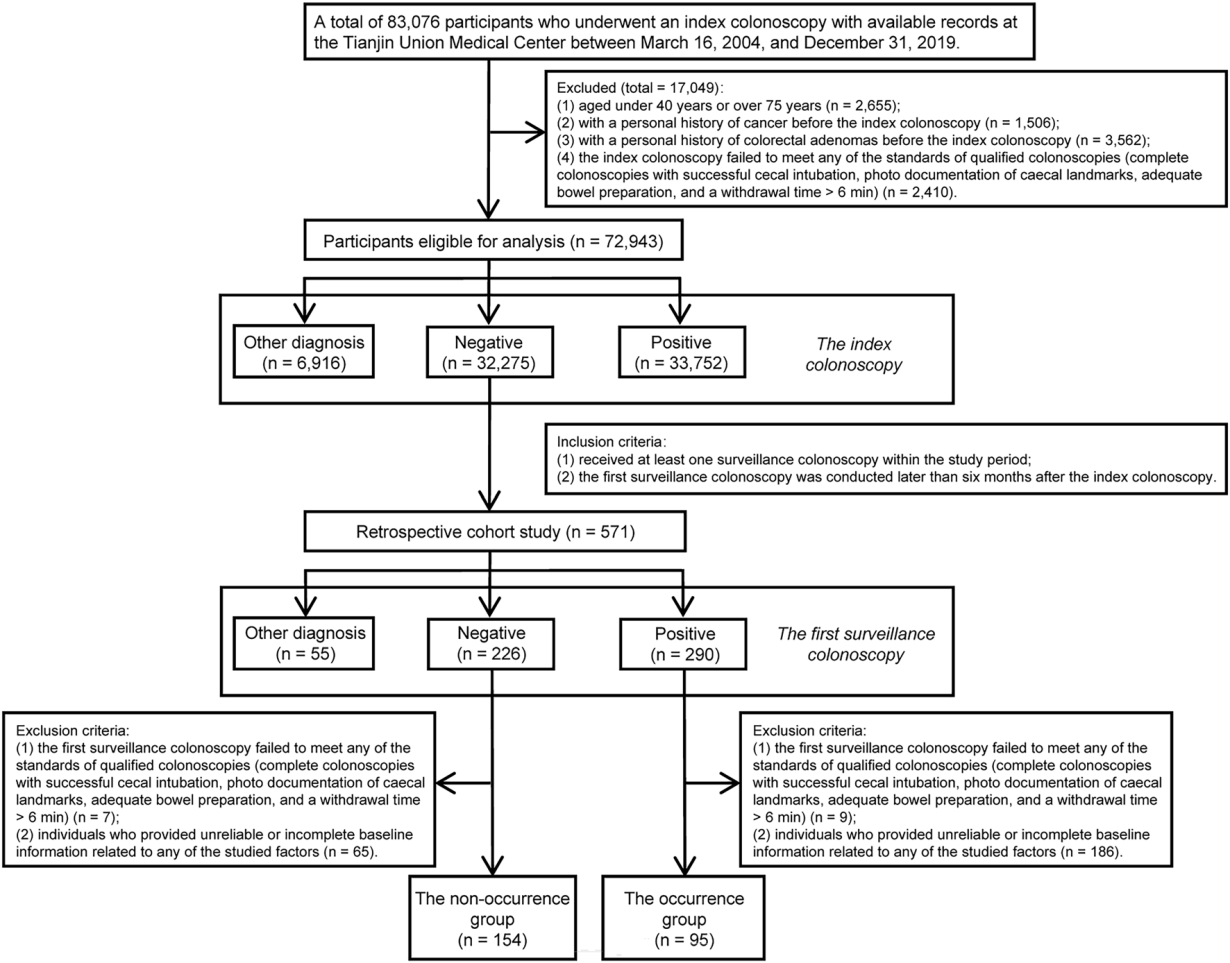
Flow diagram depicting the procedure of subject inclusion.

**Figure 2:**
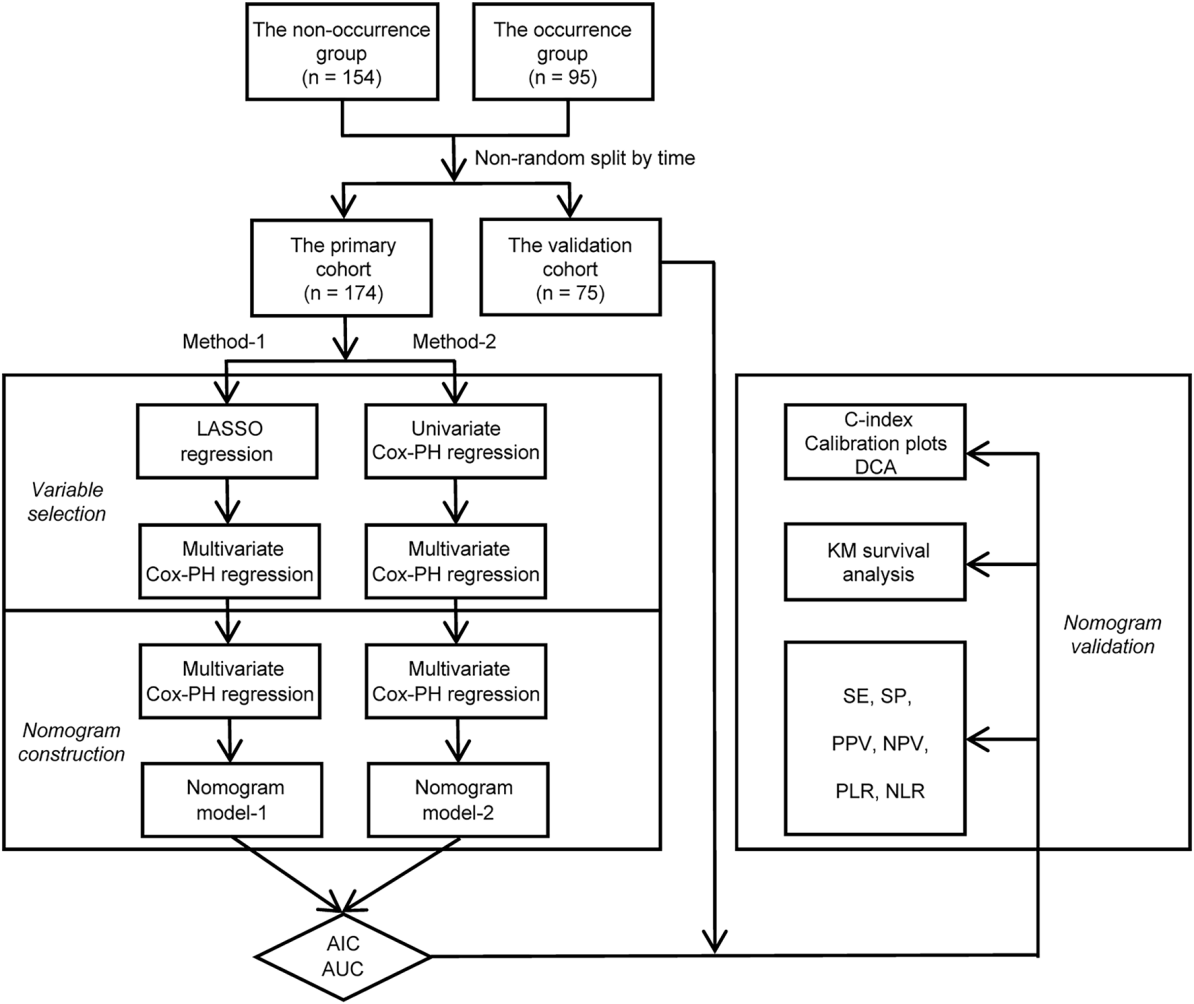
Flow diagram depicting the process of variable selection, nomogram construction and performance validation. LASSO: least absolute shrinkage and selection operator; Cox-PH: Cox proportional hazards; AIC: Akaike information criterion; AUC: area under the curve; C-index: concordance index; DCA: decision curve analysis; KM: Kaplan–Meier; SE: sensitivity; SP: specificity; PPV: positive predictive value; NPV: negative predictive value; PLR: positive likelihood ratio; NLR: negative likelihood ratio.

The procedure of subject inclusion is shown in Fig. 1. The process of variable selection, nomogram construction and performance validation is depicted in Fig. 2. According to meticulous inclusion and exclusion criteria, 249 eligible participants were enrolled and divided into two independent cohorts (Fig. 1). Participants who underwent an index colonoscopy between September 29, 2004, and May 6, 2016, were included in the primary cohort (n = 174) to construct the nomogram, and those who underwent an index colonoscopy between May 11, 2016, and April 10, 2019, were treated as the validation cohort (n = 75) to appraise model performance. At a median (range) follow-up time of 25.40 months (6.07–144.47), 38.15% (95 of 249) of the subjects developed colorectal adenomas. The 12-month, 24-month, and 36-month AFS percentages were 93.57%, 81.93% and 75.90%, respectively.

The demographic and clinical characteristics of subjects in the primary cohort and the validation cohort were demonstrated in Table 1. In the primary cohort, 58.05% (n = 101) of the subjects were female, with a median (IQR) age of 61.134 (58.579, 64.411) years, and a median (IQR) BMI of 23.931 (22.656, 25.952) kg/m^2^. Moreover, 14.94% (n = 26) of them were current or past smokers, and 14.94% (n = 26) of them drank occasionally, frequently or even daily. Additionally, 43.68% (n = 76) of them shared the common feature of physical inactivity, and 12.07% (n = 21) of them had a family history of CRC in FDR. The percentages of individuals who had a personal history of chronic constipation, chronic diarrhea, and chronic appendicitis or appendectomy were 29.89% (n = 52), 28.74% (n = 50) and 10.34% (n = 18), respectively. Notwithstanding a temporal disconnect, the characteristics of subjects in the validation cohort were similar to those of subjects in the primary cohort. Only age (*p* < 0.0001) and physical activity (*p* = 0.003) were significantly different between the two cohorts. The characteristics of subjects in the occurrence group and the non-occurrence group within each cohort were exhibited in Supplementary Table S1.

**Table 1:** Baseline information of demographic and clinical characteristics of subjects in the primary cohort and the validation cohort.

Variables	The primary cohort	The validation cohort	*p-*Value
	(n = 174)	(n = 75)	
Age (median (IQRs))	61.134 (58.579, 64.411)	55.586 (51.737, 60.864)	< 0.0001
BMI (median (IQRs))	23.931 (22.656, 25.952)	23.225 (22.231, 25.064)	0.094
Gender (n (%))			0.765
Female	101 (58.05)	42 (56.00)	
Male	73 (41.95)	33 (44.00)	
Smoking status (n (%))			0.324
Never	148 (85.06)	60 (80.00)	
Current or past	26 (14.94)	15 (20.00)	
Alcohol consumption (n (%))			0.324
Never	148 (85.06)	60 (80.00)	
Occasional, frequent or even daily	26 (14.94)	15 (20.00)	
Physical activity (n (%))			0.003
Physical inactivity	76 (43.68)	48 (64.00)	
Regular activity	98 (56.32)	27 (36.00)	
Family history of CRC in FDR (n (%))			0.752
No	153 (87.93)	67 (89.33)	
Yes	21 (12.07)	8 (10.67)	
History of chronic constipation (n (%))			0.465
No	122 (70.11)	56 (74.67)	
Yes	52 (29.89)	19 (25.33)	
History of chronic diarrhea (n (%))			0.924
No	124 (71.26)	53 (70.67)	
Yes	50 (28.74)	22 (29.33)	
History of chronic appendicitis or appendectomy (n (%))			0.098
No	156 (89.66)	72 (96.00)	
Yes	18 (10.34)	3 (4.00)	

Continuous variables were compared using the Wilcoxon–Mann–Whitney test. Categorical variables were compared using the Pearson Chi-square test. All tests were two-sided.

*IQR* interquartile range, *BMI* body mass index, *CRC* colorectal cancer, *FDR* first-degree relative.

### Identification of predictive factors and construction of nomogram

#### Method-1

**Figure 3:**
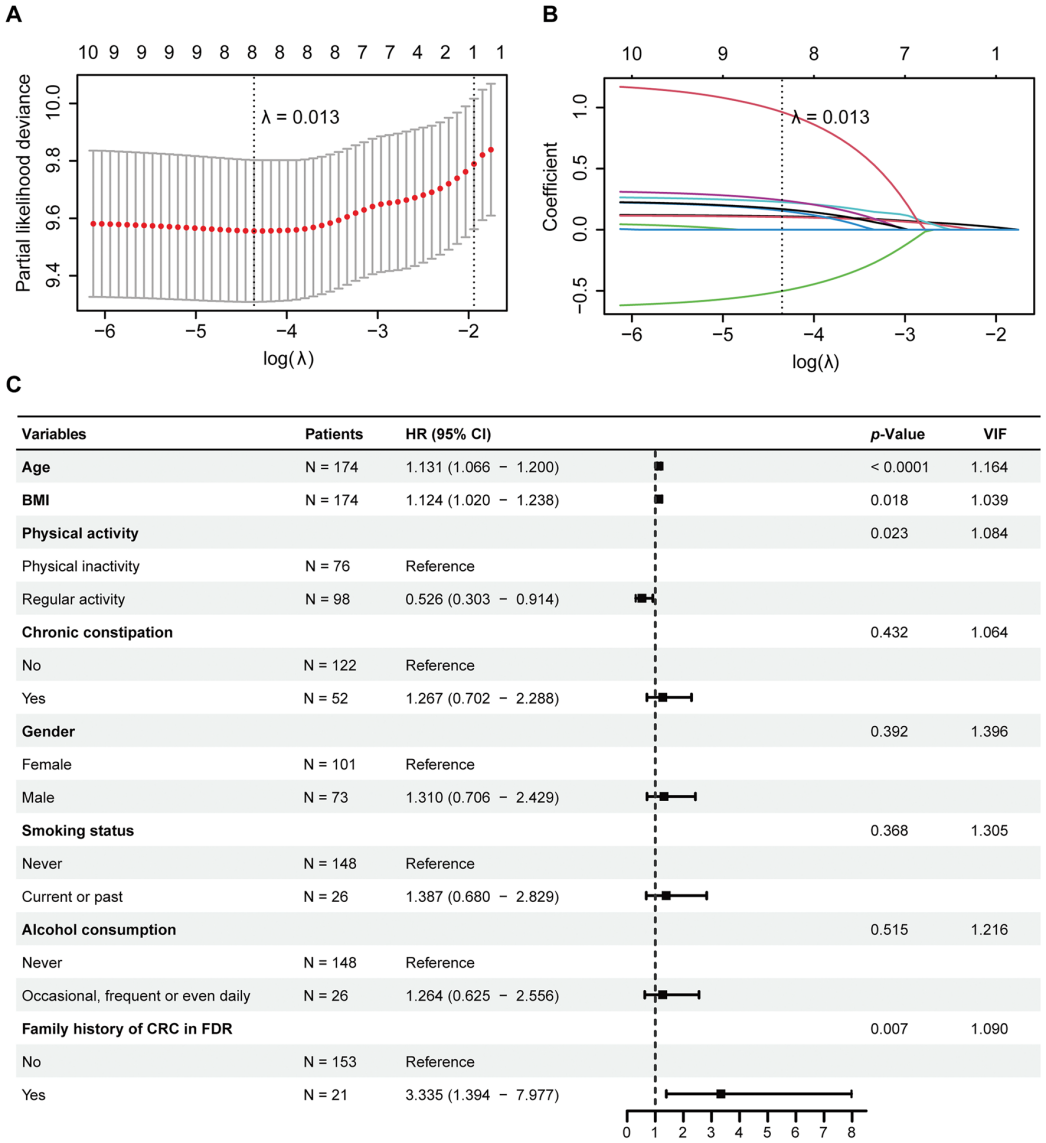
Variable selection through the LASSO-Cox regression for predicting risks of colorectal adenoma occurrence based on the primary cohort. (**A**) Tuning parameter (λ) selection of deviance in the LASSO regression analysis. The red dot denotes the CVM, and the gray line stands for the SE of CVM. (**B**) LASSO coefficient profiles of ten candidate variables. Each curve in different color signifies the trajectory of the coefficient of each variable. (**C**) A forest plot exhibiting results of the multivariate Cox-PH regression analysis. CVM: mean cross-validated error; SE: standard error; HR: hazard ratio; CI: confidence interval; VIF: variance inflation factor; BMI: body mass index; CRC: colorectal cancer; FDR: first-degree relatives.

**Figure 4:**
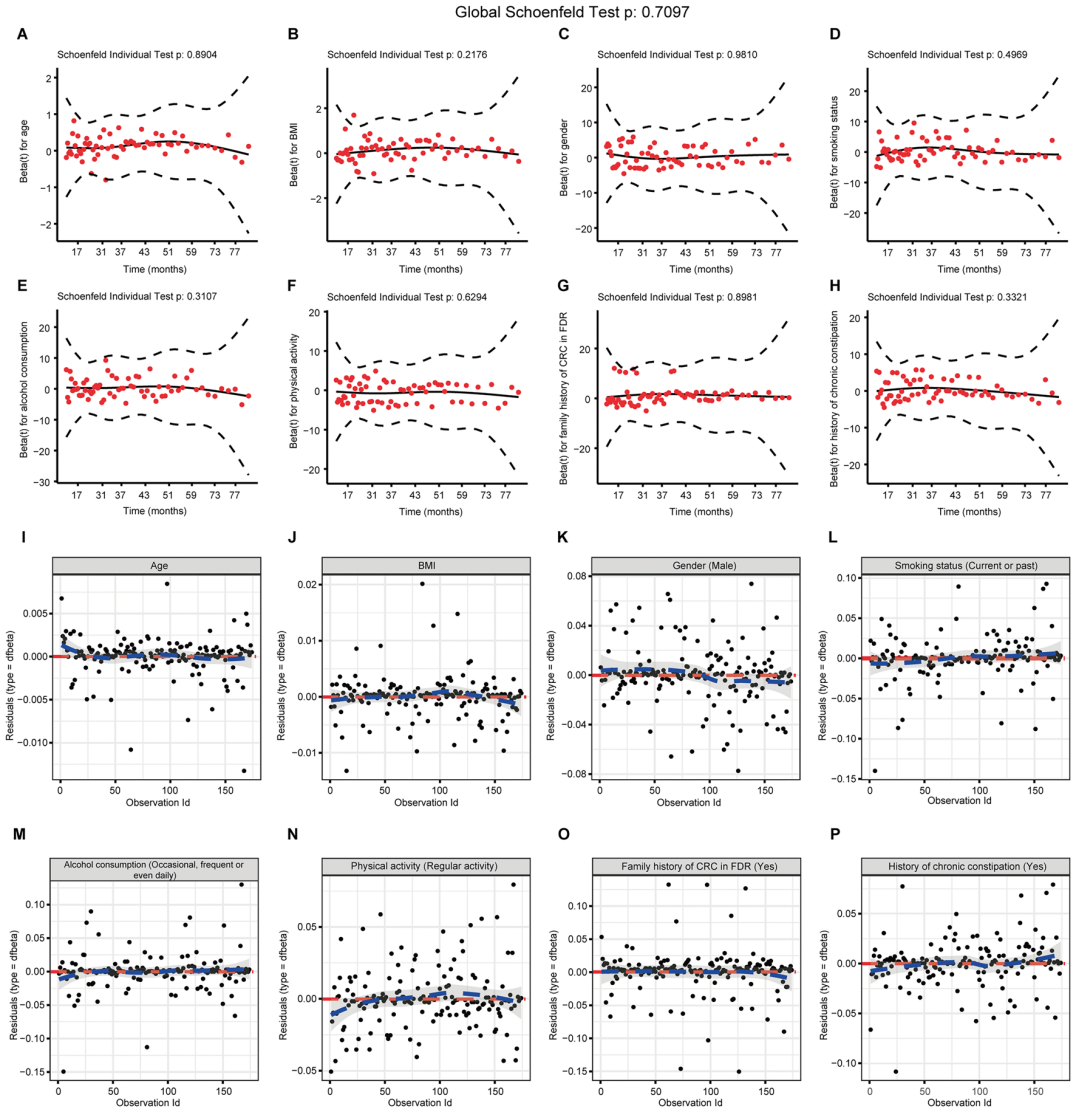
Schoenfeld’s individual and global test and deviance residual were conducted to appraise whether the eight covariates selected using the LASSO regression were associated with time before performing the multivariate Cox-PH regression analysis. (**A**–**H**) Plots of the scaled Schoenfeld residuals against the changed time. The solid line represents a smoothing spline fit to the plot, and the dashed lines represent a ± 2-SE band around the fit. Considerable departures from the horizontal line indicate nonproportional hazards. (**I**–**P**) Index graphs of dfbeta for the Cox-PH regression of AFS. The graphs comparing the magnitudes of the largest dfbeta values with the regression coefficients uncover that there exist no greatly influential individual observations. AFS: adenoma-free survival.

**Figure 5:**
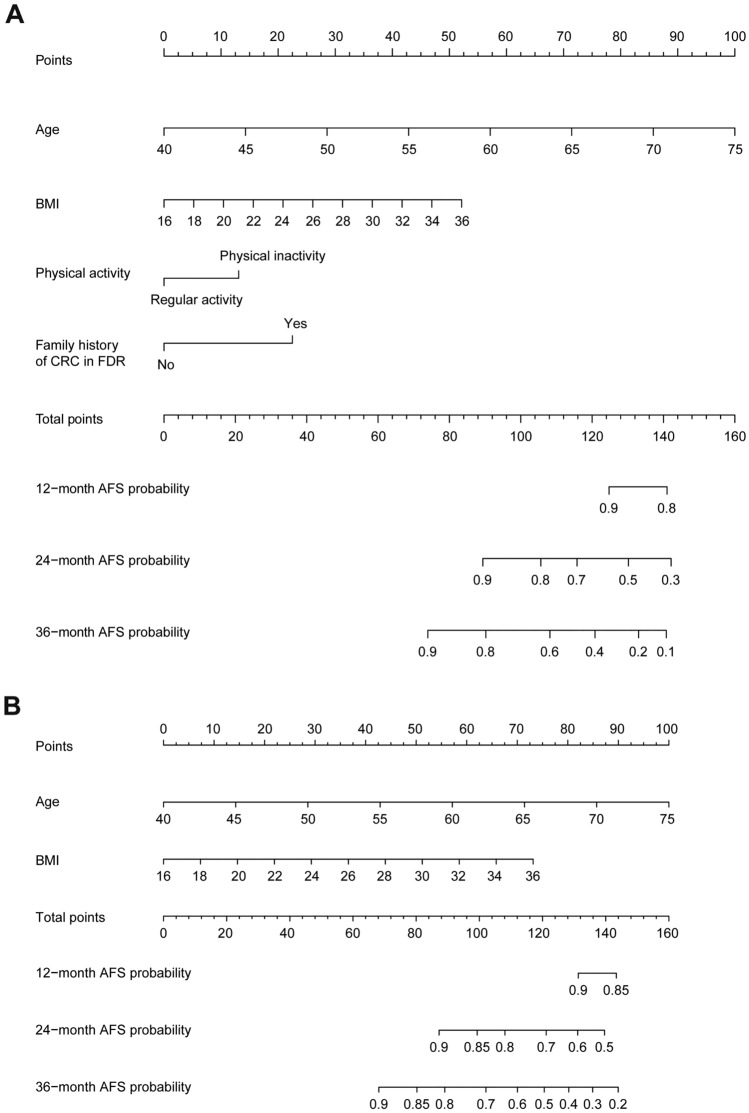
Construction of two nomogram models for predicting 12-month, 24-month, and 36-month AFS probabilities.

In the LASSO regression analysis based on tenfold cross-validation (Fig. 3A,B), a λ value of 0.013, which corresponded with a log(λ) of −4.360, was chosen according to the minimum criteria. Eight variables with non-zero coefficients were retained, including baseline age, BMI, physical activity, history of chronic constipation, gender, smoking status, alcohol consumption, and family history of CRC in FDR. As shown in Fig. 3C, the multivariate Cox-PH regression analysis disclosed that among these eight variables, age (hazard ratio [HR] = 1.131, 95% confidence interval [CI] = 1.066–1.200, *p* < 0.0001), BMI (HR = 1.124, 95% CI = 1.020–1.238, *p* = 0.018), physical activity (HR = 0.526, 95% CI = 0.303–0.914, *p* = 0.023) and family history of CRC in FDR (HR = 3.335, 95% CI = 1.394–7.977, *p* = 0.007) were independent predictors for the development of colorectal adenomas, while other parameters demonstrated no significant association (*p* > 0.05). No VIF ≥ 5 was detected (Fig. 3C), which excluded the possibility of multicollinearity among variables and justified our use of the multivariate Cox-PH regression. As demonstrated in Fig. 4A–H, the results of Schoenfeld’s individual and global test indicated no violation of the PH assumption (*p* > 0.05), proving that none of the covariates were time-varying. The results of deviance residual also uncovered that none of the individual observations were highly influential (Fig. 4I–P). Subsequently, we incorporated these four variables into Nomogram-1 (Fig. 5A) formulated using the multivariate Cox-PH regression, the result of which was demonstrated in Table 2. No VIF ≥ 5 (Table 2) or breach of the PH assumption (*p* > 0.05, Supplementary Figure S1A–D) was detected, and there existed no greatly influential individual observations (Supplementary Figure S1E–H). The AIC, 24-month AUC, 30-month AUC and 36-month AUC values were calculated as 502.451, 0.657, 0.638, and 0.642 respectively.

**Table 2:** Results of the multivariate Cox-PH regression analysis for construction of two nomogram models.

Variables	Nomogram model-1			Nomogram model-2		
	HR^a^ (95% CI)	*p-*Value	VIF	HR^b^ (95% CI)	*p*-Value	VIF
Age	1.141 (1.078–1.207)	< 0.0001	1.106	1.108 (1.053–1.166)	< 0.0001	1.000
BMI	1.128 (1.029–1.237)	0.010	1.008	1.141 (1.040–1.251)	0.005	1.000
Physical activity		0.029			–	
Physical inactivity	Reference		Reference	–		–
Regular activity	0.547 (0.317–0.942)		1.062	–		–
Family history of CRC in FDR		0.016			–	
No	Reference		Reference	–		–
Yes	2.825 (1.214–6.572)		1.060	–		–

^a^HRs were adjusted for baseline age, BMI, physical activity and family history of CRC in FDR.

^b^HRs were adjusted for baseline age and BMI.

*Cox-PH* Cox proportional hazards, *HR* hazard ratio, *CI* confidence interval, *VIF* variance inflation factor.

#### Method-2

The univariate Cox-PH regression analysis elucidated that older age (HR = 1.099, 95% CI = 1.048–1.153, *p* = 0.001), higher BMI (HR = 1.147, 95% CI = 1.038–1.269, *p* = 0.007) and male gender (HR = 1.743, 95% CI = 1.073–2.832, *p* = 0.025) were significantly associated with an elevated risk of developing colorectal adenomas (Table 3). The results of Schoenfeld’s tests and deviance residual were shown in Supplementary Figure S2. The multivariate Cox-PH regression analysis further disclosed that among the three variables, only age (HR = 1.102, 95% CI = 1.047–1.161, *p* < 0.0001, Table 3) and BMI (HR = 1.133, 95% CI = 1.031–1.246, *p* = 0.010) were independent predictors for the development of colorectal adenomas, while other parameters demonstrated no significant association (*p* > 0.05). No VIF ≥ 5 was detected (Table 3), and the results of Schoenfeld’s test and deviance residual suggested no breach of the PH assumption (*p* > 0.05, Supplementary Figure S3A–C) or greatly influential individual observations (Supplementary Figure S3D–F). Subsequently, we incorporated these two variables into Nomogram-2 (Fig. 5B) formulated using multivariate Cox-PH regression, the result of which was demonstrated in Table 2. No VIF ≥ 5 (Table 2) or breach of the PH assumption (*p* > 0.05, Supplementary Figure S4A,B) was detected, and there existed no greatly influential individual observations (Supplementary Figure S4C,D). The AIC, 24-month AUC, 30-month AUC and 36-month AUC values were calculated as 506.608, 0.590, 0.603 and 0.605 respectively.

**Table 3:** Variable selection through the univariate/multivariate Cox-PH regression analysis for predicting risks of colorectal adenoma occurrence based on the primary cohort.

Variables	The univariate Cox-PH regression analysis		Variables	The multivariate Cox-PH regression analysis		
	HR (95% CI)	*p-*Value		HR^a^ (95% CI)	*p-*Value	VIF
Age	1.099 (1.048–1.153)	0.001	Age	1.102 (1.047–1.161)	< 0.0001	1.040
BMI	1.147 (1.038–1.269)	0.007	BMI	1.133 (1.031–1.246)	0.010	1.003
Gender		0.025	Gender		0.272	
Female	Reference		Female	Reference		Reference
Male	1.743 (1.073–2.832)		Male	1.325 (0.802–2.189)		1.043
Smoking status		0.197	Smoking status		–	
Never	Reference		Never	–		–
Current or past	1.481 (0.816–2.691)		Current or past	–		–
Alcohol consumption		0.077	Alcohol consumption		–	
Never	Reference		Never	–		–
Occasional, frequent or even daily	1.683 (0.945–3.000)		Occasional, frequent or even daily	–		–
Physical activity		0.438	Physical activity		–	
Physical inactivity	Reference		Physical inactivity	–		–
Regular activity	0.822 (0.500–1.349)		Regular activity	–		–
Family history of CRC in FDR		0.255	Family history of CRC in FDR		–	
No	Reference		No	–		–
Yes	1.590 (0.716–3.529)		Yes	–		–
History of chronic constipation		0.495	History of chronic constipation		–	
No	Reference		No	–		–
Yes	1.203 (0.707–2.047)		Yes	–		–
History of chronic diarrhea		0.586	History of chronic diarrhea		–	
No	Reference		No	–		–
Yes	0.861 (0.502–1.477)		Yes	–		–
History of chronic appendicitis or appendectomy		0.704	History of chronic appendicitis or appendectomy		–	
No	Reference		No	–		–
Yes	1.179 (0.504–2.759)		Yes	–		–

^a^HRs were adjusted for baseline age, BMI and gender.

Consequently, we chose Nomogram-1, which harbored both lower AIC and higher AUC values than Nomogram-2, for predicting risks of colorectal adenoma occurrence. Within Nomogram-1, points are assigned by drawing a line upward from the corresponding values to the “Points” line. The sum of these four points, plotted downward on the “Total Points” line, corresponds with predictions of 12-month, 24-month, and 36-month AFS probabilities.

### Validation of nomogram performance

The total score of these predictors was 182.504 points. The optimal cut-off value of the nomogram was 89.026 in the primary cohort and 93.581 in the validation cohort. Under the optimal cut-off value, the SE, SP, PPV, NPV, PLR, and NLR were 69.70%, 57.41%, 50.00%, 75.61%, 1.636 and 0.528 respectively in the primary cohort, and were 31.03%, 86.96%, 60.00%, 66.67%, 2.379, and 0.793 respectively in the validation cohort.

**Figure 6:**
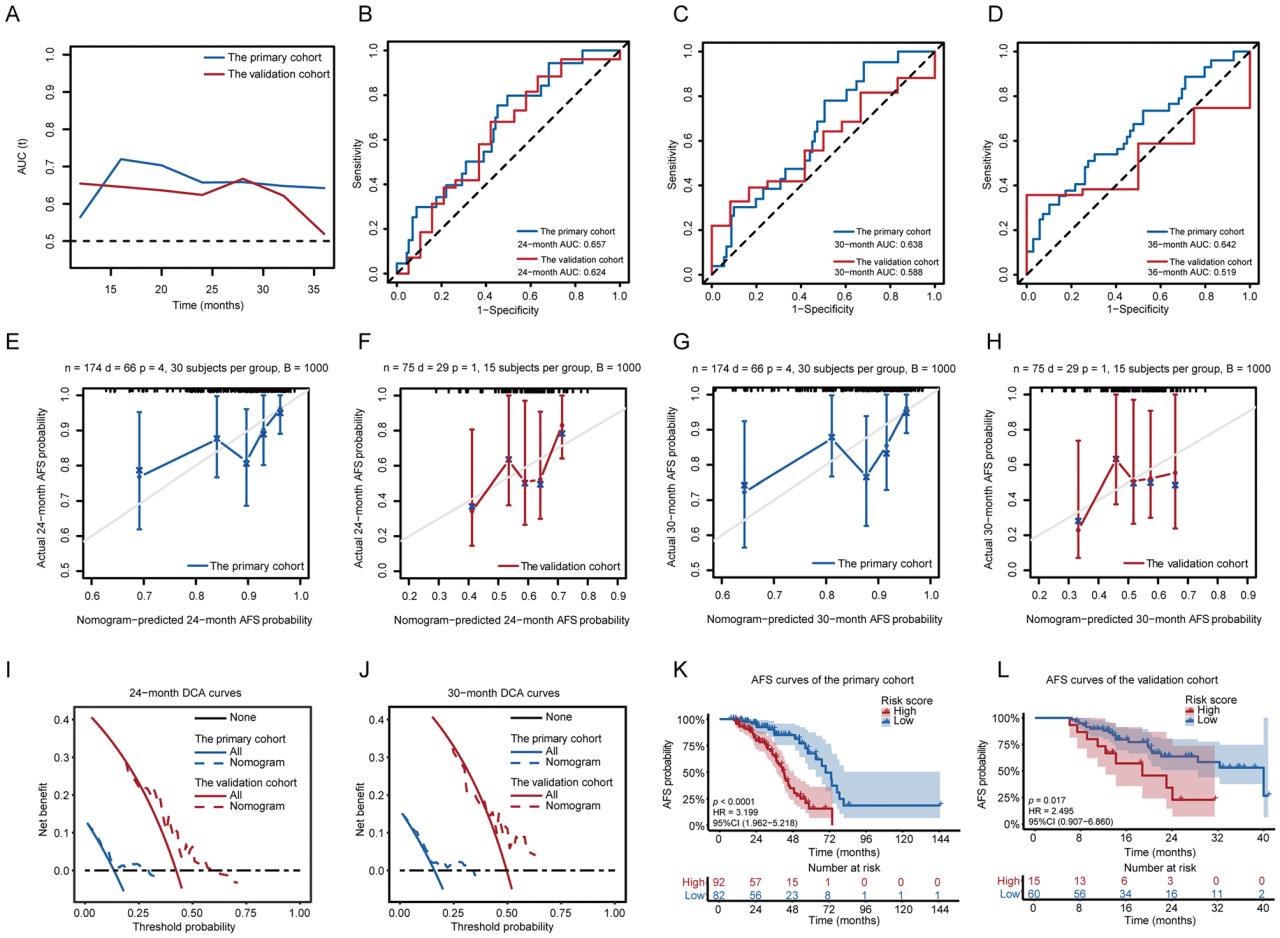
The predictive accuracy, discriminatory ability, clinical utility, and risk stratification capacity of the nomogram for predicting AFS probabilities were evaluated using the tROC curves, calibration plots, DCA curves, and KM survival curves. (**A**) The tROC curves indicated stable predictive accuracy of the nomogram over time in the primary cohort (blue) and the validation cohort (red). (**B**–**D**) The ROC curves of the nomogram for predicting 24-month (**B**), 30-month (**C**), and 36-month (**D**) AFS probabilities based on the primary cohort (blue) and the validation cohort (red). (**E**–**H**) Calibration plots exhibited a robust pertinence between the actual probability (y-axis) and the predicted probability (x-axis) of 24-month AFS in the primary cohort (**E**) and the validation cohort (**F**). There also existed a high consistency between the actual probability (y-axis) and the predicted probability (x-axis) of 30-month AFS in the primary cohort (**G**) and the validation cohort (**H**). The grey line represents the ideal fit. The blue or red line reflects the nomogram prediction, of which a closer fit to the grey line suggests better performance. (**I**,**J**) DCA of the nomogram in the primary cohort (blue) and the validation cohort (red) at 24-month (**I**) and 30-month (**J**) follow up. The black dotted line represents the screen-none scheme. The red or blue solid line represents the screen-all strategy. The red or blue dotted line represents the nomogram. (**K**,**L**) KM survival curves demonstrating the AFS probabilities in the primary cohort (**K**) and the validation cohort (**L**) with individual survival numbers and time data. tROC: time-dependent receiver operating characteristic; DCA: decision curve analysis; AUC: area under the curve.

To further evaluate the performance of Nomogram-1, the time-dependent ROC curves of the primary cohort and the validation cohort were delineated for AFS status (Fig. 6A). The C-index and bootstrapping-corrected C-index were calculated as 0.682 and 0.663 respectively for the primary cohort, indicating reliable predictive accuracy of the nomogram. The ROC curves of the primary cohort for 24-month, 30-month and 36-month AFS were shown in Fig. 6B–D. Calibration plots exhibited a robust pertinence between the actual probability (y-axis) and the predicted probability (x-axis) of 24-month AFS in the primary cohort and the validation cohort. There also existed a high consistency between the actual probability (y-axis) and the predicted probability (x-axis) of 30-month AFS in the primary cohort and the validation cohort (Fig. 6E–H). DCA for 24-month and 30-month AFS also manifested that applying our nomogram to identify patients who developed colorectal adenomas after negative index colonoscopy had an edge over the scheme of ‘‘surveillance colonoscopy for no patients’’ and the strategy of ‘‘surveillance colonoscopy for all patients’’, suggesting great clinical utility of the nomogram in both cohorts (Fig. 6I,J). To assess the risk stratification ability of Nomogram-1, participants were divided into the high-risk group and the low-risk group based on the optimal cut-off value of risk scores in each cohort. The KM survival curves with individual survival numbers and time data were delineated in Fig. 6K–L. Log-rank tests revealed a significantly lower proportion of adenoma-free subjects in the high-risk group compared with that in the low-risk group in the primary cohort (Fig. 6K, *p* < 0.0001) and the validation cohort (Fig. 6L, *p* = 0.017).

## Discussion

In this retrospective cohort study, we evaluated the pertinence between baseline patient characteristics involving modifiable lifestyle factors and adenoma occurrence risks based on high-risk populations with negative index colonoscopy. Four factors encompassing baseline age, BMI, physical activity and family history of CRC in FDR were selected to construct a nomogram for predicting AFS probabilities. Our nomogram had great discriminatory ability for predicting AFS rates, with a C-index of 0.682 and a bootstrapping-corrected C-index of 0.663 in the primary cohort. Additionally, the calibration curves illustrated that the nomogram-predicted probability was closely aligned with the actual probability in both cohorts. The DCA curves and KM survival curves also illuminated that our model had sufficient net benefit for clinical application and convincing risk stratification ability.

Mounting studies have investigated putative risk factors for colorectal adenoma occurrence. Population-based national cancer screening programs emphasized that the detection rates for non-advanced adenomas, advanced neoplasms, and any neoplasms all increased with age^7^. A Korean study involving asymptomatic participants who underwent screening colonoscopies from January 2006 to June 2009 uncovered that the prevalence of advanced adenomas escalated from 0.60% among participants ages 30 to 39 to 7.40% among those ages 70–79^40^. Moreover, a 5-unit increase in BMI is correlated with a 19% increased risk of colorectal adenomas as demonstrated by a comprehensive meta-analysis^32^. Physical activity, especially leisure and sport activity, was corroborated to protect against colorectal adenomas^19^. A multinational, prospective colonoscopy study which involved 16 Asia–Pacific regions from December 2011 to December 2013 disclosed that subjects without a family history of CRC in FDR were less likely to have colorectal adenomas than those with at least one FDR affected (adjusted odds ratio = 1.31–1.92)^34^. In line with previous literature, older age and higher BMI were identified as independent risk factors for the development of colorectal adenomas by both the LASSO-Cox regression and the univariate/multivariate regression in the present study. Physical activity and family history of CRC in FDR were also independently associated with colorectal adenoma occurrence according to the LASSO-Cox regression analysis.

Moreover, the relationship between other studied factors and colorectal adenoma occurrence has also been systematically interrogated. Recent studies manifested that the prevalence and incidence of colorectal adenomas in women were significantly lower than those in men^30,33^, possibly owing to the lower colonoscopy completion rate among women^41^, who prefer more convenient and non-invasive screening approaches such as computed tomography colonography^42^ and multitarget stool DNA testing^43^. Additionally, previous studies disclosed that the prevalence of colorectal polyps increased by 3.40 times in current smokers as compared to never smokers^44^. Dose-response relations were also detected among the pack-years of smoking, the duration of smoking, the daily number of cigarettes smoked, and risks of colorectal adenomas^45^. DNA hypomethylation in the normal rectal mucosa^46^, genetic variants in mismatch repair enzymes^47^, and polymorphisms in tobacco-carcinogen-metabolizing pattern^48^ might be parts of underlying mechanisms. Besides, recent meta-analysis underscored the positive correlation between alcohol consumption and risks of colorectal adenomas^18^. Proposed pathophysiological mechanisms involve the effects of acetaldehyde and reactive oxygen species, induction of cytochrome P 4502E1, as well as nutritional deficiency^49^. Furthermore, higher prevalence and incidence of CRC and benign colorectal neoplasms were observed among patients with chronic constipation compared with matched chronic constipation-free patients^35^. Chronic diarrhea was also considered as an independent risk factor for colorectal ademoma occurrence^31^, presumably owing to structural and functional disruption of the colonic epithelium caused by increased levels of secondary bile acids through oxidative damage to DNA, inflammation, and enhanced cell proliferation^50^. A history of chronic appendicitis or appendectomy had a higher sensitivity than a history of adenomatous polyps for surveillance of colorectal adenomas^36^. However, no significant association between each of these variables and risks of colorectal ademoma occurrence was found in the present study, which might be attributed to different study designs and effects of small sample size.

Length of screening intervals and numbers of necessary examinations are crucial parameters for CRC screening. Considering the astronomical economic and healthcare burden of CRC screening and surveillance, greater efforts are warranted to ensure that colonoscopy services are delivered to qualified patients at proper intervals^10^. Hitherto, there is a lack of adequate and clear guidance on the timing of follow-up colonoscopies after negative index colonoscopy. For asymptomatic average-risk individuals whose index colonoscopy detected no polyps or detected merely hyperplastic polyps in the rectum and sigmoid colon sized < 10 mm, current guidelines generally recommend them to undergo surveillance colonoscopies after ten years^10,29,51^. As for high-risk adults with negative index colonoscopy, surveillance intervals recommended by current guidelines are broad and diverse, and most of them merely consider risk factors including family history of CRC in FDR, inflammatory bowel disease and hereditary syndromes^51–54^ and tend to ignore modifiable lifestyle factors. Nevertheless, familial and hereditary factors and lifestyle-related patterns often co-exist and interact in the etiology of colorectal neoplasms^55^, and the synergistic effects of multiple lifestyle factors are non-negligible in the scheduling of surveillance colonoscopies in real life^56,57^. This research gap may give rise to inaccurate risk predictions and inappropriate surveillance recommendations, thus raising the likelihood of  over-use or under-use of surveillance colonoscopies. Over-use of colonoscopies is notorious for increased risks of adverse events, extra financial burdens, and deprived opportunities for under-screened populations to undergo adequate colonoscopy service, while under-use of colonoscopies is detrimental to the accuracy and timeliness of CRC diagnosis^10^. Notably, our model can provide accurate and convenient predictions of 24-month, 30-month and 36-month AFS probabilities for high-risk individuals with negative index colonoscopy based on their lifestyle patterns, thus optimizing the timing of follow-up colonoscopies for effective prevention of colorectal adenomas and CRC.

Specific limitations deserve careful attention during interpretation of our results. First, considering the retrospective design of our study and the subjective nature of lifestyle patterns, the inherent recall bias was inevitable. Second, conducted in a single center based on a relatively small sample of participants in Tianjin, our study was subjected to unavoidable selection bias, and the generalizability of our nomogram would be compromised correspondingly. Multicenter, large-scale, prospective investigations with external validation data from other cities and nations are warranted to polish our model in the future. Third, although we used the multivariate Cox-PH regression to control confounding factors, other factors such as dietary factors including intakes of red meat or processed meat, fiber and calcium, and some clinical features encompassing use of insulin, C-peptide and aspirin, were not controlled because related information was incomplete or missing. The most highlighted strength of our study is that the studied covariates are usually available from electronic medical records, thus ensuring the feasibility and convenience of applying our nomogram model to clinical practice. Furthermore, we excluded subjects whose first surveillance colonoscopy was conducted within six months after negative index colonoscopy or failed to meet any of the standards of qualified colonoscopies, so as to mitigate the impacts of missing adenomas on our analysis.

## Conclusions

To summarize, we incorporated four baseline patient characteristics including age, BMI, physical activity and family history of CRC in FDR, which were selected using the LASSO-Cox regression analysis, into a nomogram model for predicting risks of colorectal adenoma occurrence among high-risk populations with negative index colonoscopy. Our model not only had good predictive accuracy, discriminatory ability and clinical application value, but also effectively stratified individuals with different risk scores into different risk tiers of survival outcomes. Our model will add a new dimension to the implementation of individualized prevention against colorectal adenomas and CRC.

### Supplementary Information

Supplementary Information.

## Data Availability

The datasets generated during and/or analysed during the current study are not publicly available due to protection of patients’ privacy but are available from the corresponding author on reasonable request.
