# Supplementary material for: Derivation and validation of a nomogram incorporating modifiable lifestyle factors to predict development of colorectal adenomas after negative index colonoscopy
Source: Sci Rep. 2024 May 21;14:11633. doi: 10.1038/s41598-024-62348-w (PMC11109095; doi:10.1038/s41598-024-62348-w)
Supplement: Supplementary file 1 — Supplementary Information. [file 41598_2024_62348_MOESM1_ESM.pdf]

Supplementary Material

Derivation and validation of a nomogram incorporating modifiable lifestyle factors to predict development of colorectal adenomas after negative index colonoscopy

Mingqian Yu[1,2,+], Yiben Ouyang[2,+], Zhen Yuan[2,+], Shuyuan Wang[2,+], Wenwen Pang[5], Suying Yan[1,3], Xinyu Liu[4], Wanting Wang[1,3], Ben Yi[1,3], Qiurong Han[1,3], Yao Yao[1,3], Yanfei Liu[1,3], Tianhao Chu[1,3], Zhiqiang Feng[1,3], Qinghuai Zhang[1,6,7], Xipeng Zhang[1,6,7,*], Chunze Zhang[1,6,7,*]

[1]Department of Colorectal Surgery, Tianjin Union Medical Center, Tianjin, China.

[2]School of Medicine, Nankai University, Tianjin, China.

[3]School of Integrative Medicine, Tianjin University of Traditional Chinese Medicine, Tianjin, China.

[4]Tianjin Medical University, Tianjin, China.

[5]Department of clinical laboratory, Tianjin Union Medical Center, Tianjin, China.

[6]The Institute of Translational Medicine, Tianjin Union Medical Center, Tianjin, China.

[7]Tianjin Institute of Coloproctology, Tianjin, China.

*** Correspondence:**
Chunze Zhang, Department of Colorectal Surgery, Tianjin Union Medical Center, Tianjin, China.

E-mail: chunze.zhang@nankai.edu.cn;

Xipeng Zhang, Department of Colorectal Surgery, Tianjin Union Medical Center, Tianjin, China.

E-mail: xipengzhangtj@163.com.

[+]these authors contributed equally to this work

1 Supplementary Figures and Tables

1.1 Supplementary Figures

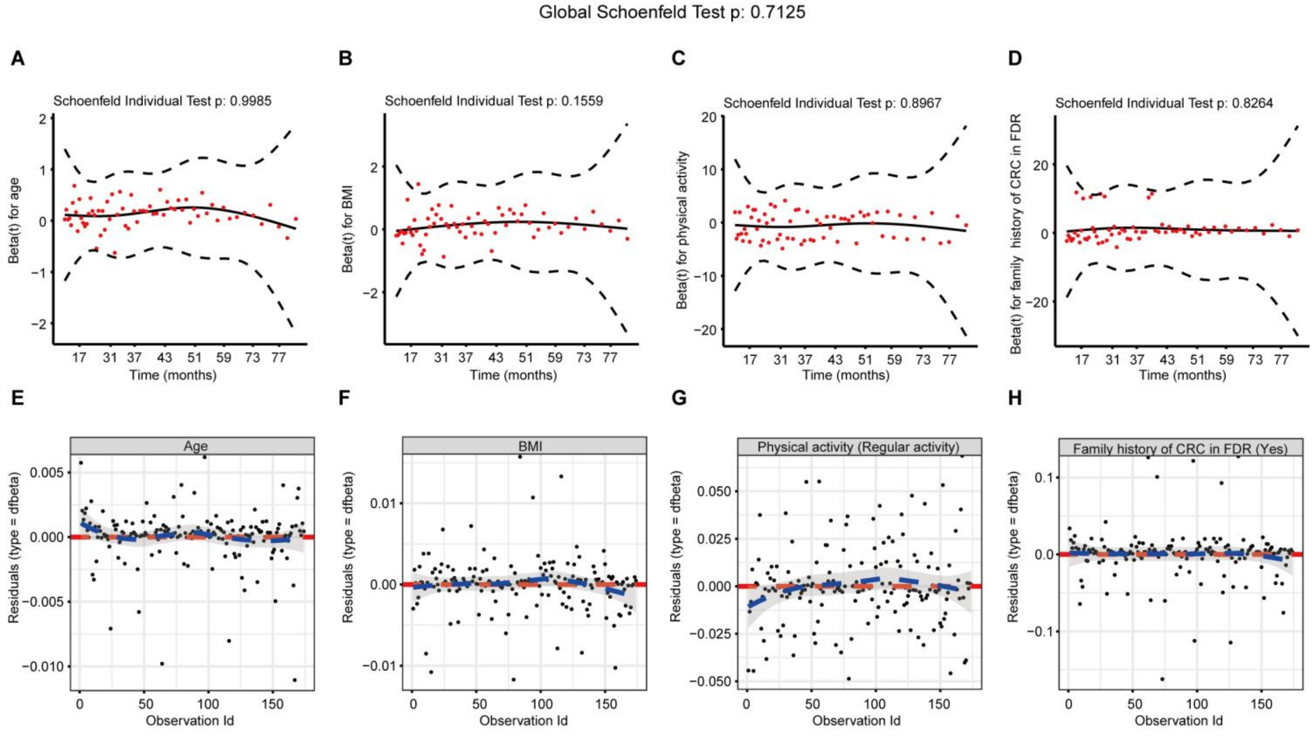

Supplementary Figure S1. Schoenfeld's individual and global test and deviance residual were utilized to estimate whether the four covariates selected using the LASSO-Cox regression analysis were time-varying before construction of Nomogram-1 using the multivariate Cox-PH regression analysis. (**A**) Plots of the scaled Schoenfeld residuals against the changed time. The solid line represents a smoothing spline fit to the plot, and the dashed lines denote a ± 2-SE band around the fit. Substantial departures from the horizontal line symbolize nonproportional hazards. (**B**) Index diagrams of dfbeta for the Cox-PH regression of AFS. The diagrams that compare the magnitudes of the largest dfbeta values to the regression coefficients unearth that none of the individual observations are greatly influential. LASSO: least absolute shrinkage and selection operator; Cox-PH: Cox proportional hazards; SE: standard error; AFS: adenoma-free survival; BMI: body mass index; CRC: colorectal cancer; FDR: first-degree relative.

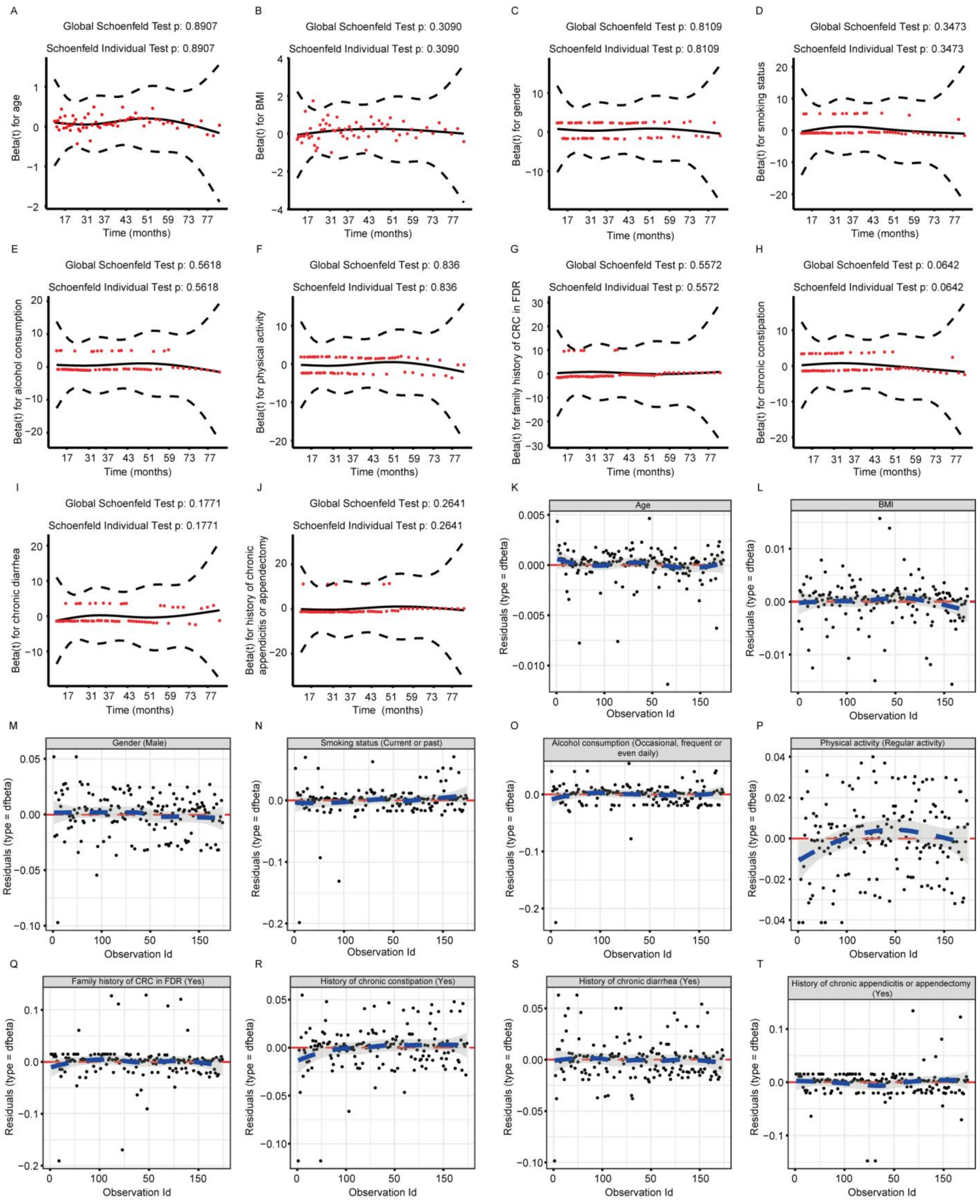

Supplementary Figure S2. Schoenfeld's individual and global test and deviance residual were performed to assess whether the ten candidate variables for the univariate Cox-PH regression analysis were time-dependent. (**A-J**) Plots of the scaled Schoenfeld residuals against the changed time. The solid line represents a smoothing spline fit to the plot, and the dashed lines signify a ± 2-SE band around the fit. Considerable departures from the horizontal line suggest nonproportional hazards. (**K-T**) Index graphs of dfbeta for the Cox-PH regression of AFS. The graphs which make

comparison between the magnitudes of the largest dfbeta values and the regression coefficients reveal that none of the individual observations are greatly influential.

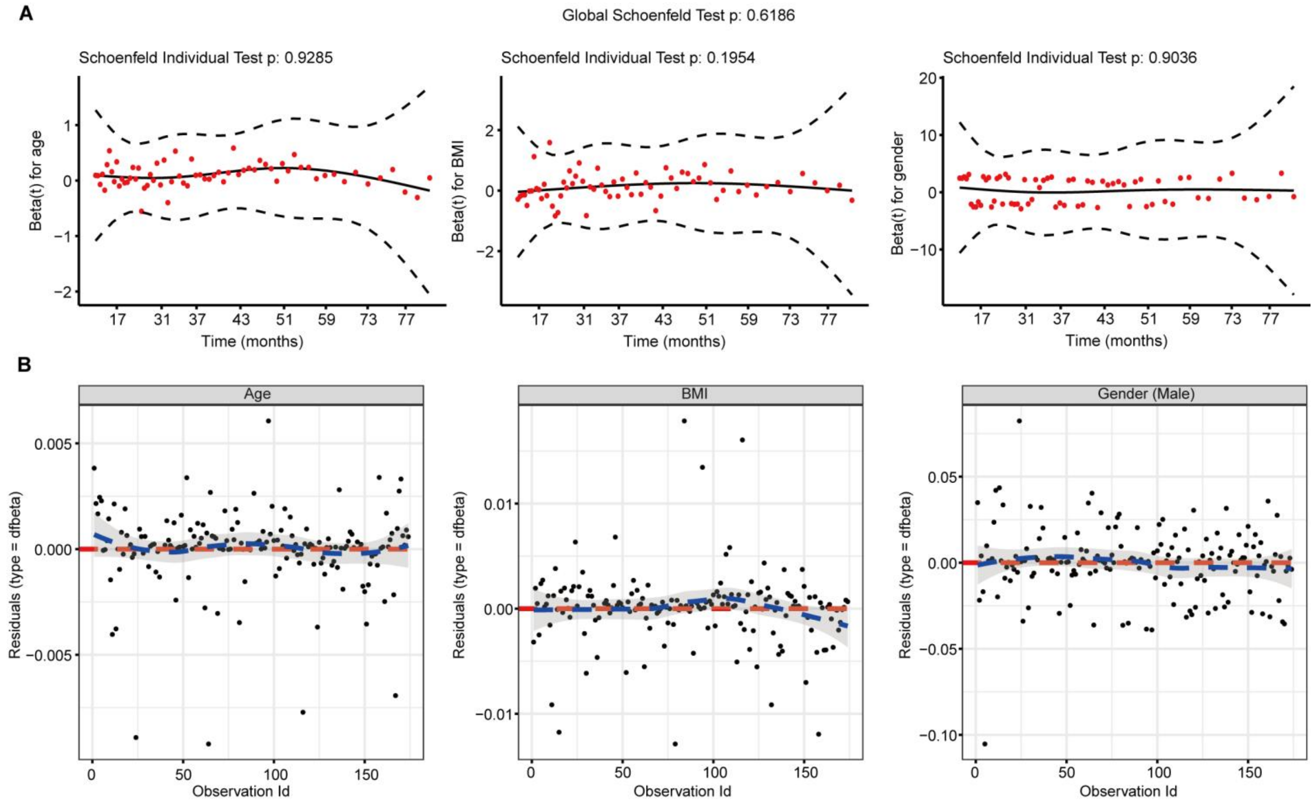

Supplementary Figure S3. Schoenfeld's individual and global test and deviance residual were utilized to estimate whether the three covariates selected using the univariate Cox-PH regression analysis were time-varying before performing the multivariate Cox-PH regression analysis. (**A**) Plots of the scaled Schoenfeld residuals against the changed time. The solid line represents a smoothing spline fit to the plot, and the dashed lines denote a ± 2-SE band around the fit. Substantial departures from the horizontal line symbolize nonproportional hazards. (**B**) Index diagrams of dfbeta for the Cox-PH regression of AFS. The diagrams which compare the magnitudes of the largest dfbeta values to the regression coefficients unearth that none of the individual observations are greatly influential.

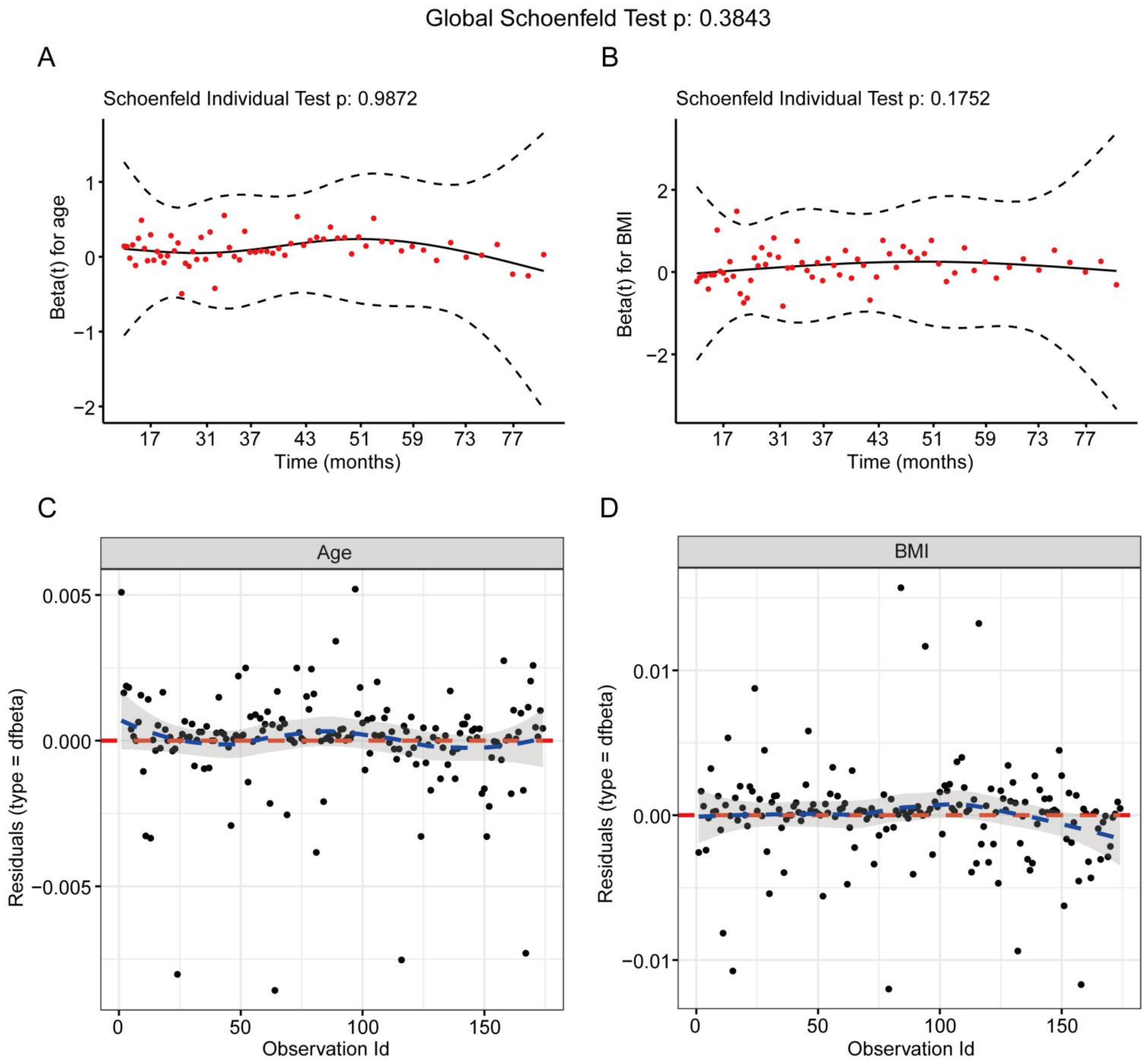

Supplementary Figure S4. Schoenfeld's individual and global test and deviance residual were utilized to estimate whether the two covariates selected using the univariate/multivariate Cox-PH regression analysis were time-varying before construction of Nomogram-2 using the multivariate Cox-PH regression. (**A**) Plots of the scaled Schoenfeld residuals against the changed time. The solid line represents a smoothing spline fit to the plot, and the dashed lines denote a ± 2-SE band around the fit. Substantial departures from the horizontal line symbolize nonproportional hazards. (**B**) Index diagrams of dfbeta for the Cox-PH regression of AFS. The diagrams which compare the magnitudes of the largest dfbeta values to the regression coefficients unearth that none of the individual observations are greatly influential.

1.2 Supplementary Tables

Supplementary Table S1. Baseline information of demographic and clinical characteristics and lifestyle patterns of subjects in the occurrence group and the non-occurrence group within both cohorts. [a] Continuous variables compared using the Wilcoxon–Mann–Whitney test. [b] Continuous variables compared using the Student's *t*-test. [c] Categorical variables compared using the Pearson Chi-square test. [d] Categorical variables compared using the continuity correction Chi-square test. All tests were two-sided, and $p < 0.05$ was considered statistically significant. IQR: interquartile range; SE: standard error; BMI: body mass index; CRC: colorectal cancer; FDR: first-degree relative.

	**The primary cohort**				**The validation cohort**		
**Variables**	**The occurrence group (n = 66)**	**The non-occurrence group (n = 108)**	***p*-Value**	**Variables**	**The occurrence group (n = 29)**	**The non-occurrence group (n = 46)**	***p*-Value**
Age (median (IQRs))	62.481 (60.342, 65.466)	60.490 (57.625, 63.549)	0.002[a]	Age (mean (mean ± SE))	60.318 (53.797, 63.910)	54.375 (50.782, 57.872)	0.011[b]
BMI (median (IQRs))	24.629 (23.314, 26.513)	23.817 (22.184, 25.786)	0.033[b]	BMI (mean (mean ± SE))	23.384 (21.613, 24.244)	23.187 (22.700, 25.155)	0.507[b]
Gender (n (%))				Gender (n (%))			
Female	31 (46.97)	70 (64.81)	0.021[c]	Female	13 (44.83)	29 (63.04)	0.122[c]
Male	35 (53.03)	38 (35.19)		Male	16 (55.17)	17 (36.96)	
Smoking status (n (%))				Smoking status (n (%))			
Never	52 (78.79)	96 (88.89)	0.070[c]	Never	21 (72.41)	39 (84.78)	0.192[c]
Current or past	14 (21.21)	12 (11.11)		Current or past	8 (27.59)	7 (15.22)	
Alcohol consumption (n (%))				Alcohol consumption (n (%))			
Never	51 (77.27)	97 (89.81)	0.024[c]	Never	21 (72.41)	39 (84.78)	0.192[c]
Occasional, frequent or even daily	15 (22.73)	11 (10.19)		Occasional, frequent or even daily	8 (27.59)	7 (15.22)	
Physical activity (n (%))				Physical activity (n (%))			

Physical inactivity	29 (43.94)	47 (43.52)	0.957[c]	Physical inactivity	14 (48.28)	34 (73.91)	0.024[c]
Regular activity	37 (56.06)	61 (56.48)		Regular activity	15 (51.72)	12 (26.09)	
Family history of CRC in FDR (n (%))				Family history of CRC in FDR (n (%))			
No	59 (89.39)	94 (87.04)	0.643[c]	No	26 (89.66)	41 (89.13)	1.000[d]
Yes	7 (10.61)	14 (12.96)		Yes	3 (10.34)	5 (10.87)	
History of chronic constipation (n (%))				History of chronic constipation (n (%))			
No	46 (69.70)	76 (70.37)	0.925[c]	No	25 (86.21)	31 (67.39)	0.068[c]
Yes	20 (30.30)	32 (29.63)		Yes	4 (13.79)	15 (32.61)	
History of chronic diarrhea (n (%))				History of chronic diarrhea (n (%))			
No	47 (71.21)	77 (71.30)	0.991[c]	No	26 (89.66)	27 (58.70)	0.004[c]
Yes	19 (28.79)	31 (28.70)		Yes	3 (10.34)	19 (41.30)	
History of chronic appendicitis or appendectomy (n (%))				History of chronic appendicitis or appendectomy (n (%))			
No	60 (90.91)	96 (88.89)	0.671[c]	No	27 (93.10)	45 (97.83)	0.681[d]
Yes	6 (9.09)	12 (11.11)		Yes	2 (6.90)	1 (2.17)	
